# Surgical Outcomes and Clinicopathological Characteristics of Patients Who Underwent Potentially Noncurative Endoscopic Resection for Gastric Cancer: A Report of a Single-Center Experience

**DOI:** 10.1155/2013/427405

**Published:** 2013-05-16

**Authors:** Hiroaki Ito, Haruhiro Inoue, Haruo Ikeda, Noriko Odaka, Akira Yoshida, Hitoshi Satodate, Manabu Onimaru, Daisuke Takayanagi, Esperanza Grace Santi, Shin-ei Kudo

**Affiliations:** Digestive Disease Center, Showa University Northern Yokohama Hospital, 35-1 Chigasakichuo, Tsuzuki-ku, Yokohama 224-8503, Japan

## Abstract

*Background*. Standard treatment of early gastric cancer (EGC) after endoscopic resection with risk factors of nodal metastases and incomplete resection is controversial. We investigated optimal management for the patients with potentially noncurative EGC after endoscopic resection. *Methods*. We retrospectively examined clinicopathological data and surgical outcomes of all patients with clinically solitary gastric adenocarcinoma who underwent curative surgery after a single peroral endoscopic resection at the Digestive Disease Center of Showa University Northern Yokohama Hospital between April 2001 and December 2012. Fisher's exact test was used for univariate analysis. For multivariate analysis, stepwise multiple linear regression was used to identify independent predictors related to lymph node metastasis and remnant of primary tumor. *Results*. A total of 41 patients were studied. Four patients (9.8%) had lymph node metastases. Primary tumors remained in 6 patients (14.6%). Only venous invasion was statistically significant to lymph node metastasis (*P* = 0.017). With respect to remnant of the primary tumor, pT1b2 tumor invasion (*P* = 0.015) and horizontal margin (*P* = 0.013) were statistically significant. *Conclusions*. Surgery with limited lymphadenectomy is recommended for tumors with venous invasion or pT1b2 tumor invasion, and additional endoscopic resection may be allowed for tumors with horizontal involvement.

## 1. Introduction

Gastric cancer is the second leading cause of cancer-related death worldwide [1]. The majority of early gastric cancer (EGC) is detected by endoscopic examination. EGC is generally recognized as a tumor with invasion confined to the mucosa or submucosa. The seventh edition of the International Union Against Cancer (UICC) TNM guidelines defines mucosal cancers as T1a and submucosal cancers as pT1b [2]. The third English edition of the Japanese Classification of Gastric Carcinoma (JCG) [3] further categorizes submucosal tumors as pT1b1 (submucosal invasion of <0.5 mm) or pT1b2 (submucosal invasion of ≥0.5 mm). Because nodal metastases are rare in pT1a tumors [4, 5], per-oral endoscopic tumor resection, such as endoscopic mucosal resection (EMR) and endoscopic submucosal dissection (ESD), has been widely performed for clinical T1a tumors. In Japan, because undifferentiated EGC has a relatively higher incidence of lymph node metastasis than does differentiated EGC, EMR and ESD have not been recommended for undifferentiated EGC [6] with the exception of tumors of <20 mm without ulceration [7, 8]. However, the depth of invasion of resected tumors is often diagnosed as pT1b or deeper. Nodal metastases occur in 2% to 9.8% of pT1b1 and 12% to 24.3% of pT1b2 tumors [9, 10]. Lymphatic and venous invasions are reported as significant risk factors of lymph node metastases [10]. Undifferentiated tumors, including mucinous carcinoma, signet-ring cell carcinoma, and poorly differentiated adenocarcinoma [10], and tumors of >3 cm [11] are also reported as risk factors for lymph node metastases. Thus, additional surgical resection including lymphadenectomy is recommended for pT1b or deeper tumors, tumors with lymphatic and venous invasions, and tumors mainly composed of undifferentiated carcinoma, although the majority of patients who undergo additional surgery may have no nodal metastasis [10].

On the other hand, additional surgical resection for tumors that were incompletely resected by endoscopic resection is controversial. JCG defines the horizontal margin (HM) and vertical margin (VM) as HM1 and VM1 (margin involvement), HM0 and VM0 (no margin involvement) or HMX and VMX (involvement of the margin cannot be assessed) [3]. Although additional endoscopic resection is recommended for HM1 tumors [12, 13] and surgical resection is recommended for VM1 tumors [12, 13], there is a possibility that no primary tumor will be present in the surgical specimen.

The aim of this study was to investigate the optimal treatment strategy for gastric cancer after endoscopic resection with risk factors of nodal metastases and incomplete resection. We estimated the relationship between clinicopathological findings of specimens from endoscopic and surgical resection and evaluated risk factors for lymph node metastases and remnant of the primary tumor. 

## 2. Patients and Methods

### 2.1. Patients

 All patients with gastric adenocarcinoma who underwent curative surgery after single per-oral endoscopic resection at the Digestive Disease Center of Showa University Northern Yokohama Hospital between April 2001 and December 2012 were retrospectively studied. The criteria for inclusion in the study were (1) a clinically solitary tumor, (2) histologically proven adenocarcinoma, (3) no prior treatment before endoscopic resection, and (4) noncurative endoscopic resection defined by the Japanese gastric cancer treatment guidelines 2010 (version 3) [14]. Patients with synchronous or metachronous malignancy were excluded. 

The formalin-fixed specimens resected by endoscopic resection were cut into multiple slices in parallel with the lesser curvature at a 5 mm interval or adequate width depending on tumor or mucosal scar size. Hematoxylin and eosin and immunohistochemical staining using antibodies of D2-40 (Dako-Japan, Tokyo, Japan) and Victoria Blue (Dako-Japan, Tokyo, Japan) were performed to detect lymphatic and venous invasions. The formalin-fixed lymph nodes were cut into two pieces, and the cut surfaces were examined. The histological diagnosis was determined by two or more examiners, including at least one board-certified pathologist, following the UICC classification and JCG. We reviewed the clinical records and histological reports to examine relationships between the histological diagnosis of specimens from endoscopic and surgical resection and the prognosis.

### 2.2. Statistical Analysis

Fisher's exact test was used for univariate analysis. Variables showing a univariate association (*P* < 0.50) were also subjected to multivariate analysis. For multivariate analysis, stepwise multiple linear regression was used to identify independent predictors related to lymph node metastasis and remnant of primary tumor. *P* values of <0.05 were considered to indicate statistical significance. Statistical analysis was performed using JMP 9.0.2 (SAS Institute, Cary, NC, USA).

The study was approved by the Institutional Review Board of Showa University, Northern Yokohama Hospital (number 1203-03). The research reported in this paper was in compliance with the Helsinki Declaration. This study was registered with the University Hospital Medical Information Network in Japan (number UMIN000007430).

## 3. Results

A total of 41 patients were eligible for inclusion in the study, including 38 males and 3 females with a mean age of 67.7 years (range, 46–83 years). The median interval between endoscopic resection and surgery was 2 months (range, 0–5 months). The median follow-up period after surgery was 23 months. No patient experienced recurrent disease after surgery.

The clinicopathological characteristics of patients are shown in Table 1. Nine and 32 of 41 patients underwent endoscopic mucosal resection and endoscopic submucosal dissection, respectively. Reasons for surgery were risk of lymph node metastasis (submucosal invasion, venous and lymphatic invasions, and undifferentiated component) and incomplete tumor resection (horizontal and vertical margin involvement). Among the 41 patients, partial, proximal, distal, and total gastrectomies were performed in 5, 9, 20, and 7 patients, respectively. 

Four (9.8%) of 41 patients had lymph node metastases in perigastric nodes. Primary tumors remained in 6 (14.6%) of the 41 patients, none of whom had metastatic nodes. Relationships among clinicopathological characteristics, nodal metastasis, and remnant of the primary tumor are summarized in Table 2. 

Four of 37 tumors with submucosal invasion (4 of 33 pT1b2 tumors), 3 of 20 with lymphatic invasion, and 4 of 18 with venous invasion had metastatic nodes. Five of 37 tumors with submucosal invasion (3 of 33 pT1b2 tumors), 4 of 20 with lymphatic invasion, 1 of 18 with venous invasion, 3 of 4 categorized as HM1, and 4 of 14 categorized as VM1 had remnants of primary tumors. 

In univariate analysis, venous invasion was the only significant factor for nodal metastasis (*P* = 0.047). Tumor size (*P* = 0.445), depth of tumor invasion (*P* = 0.404), ulceration (*P* = 0.582), main histologic type (*P* = 0.517), and lymphatic invasion (*P* = 0.284) were not significant factors for nodal metastasis. 

For remnant of the primary tumor, endoscopic procedure (*P* = 0.015) and horizontal margin (*P* = 0.001) were significant factors. Depth of tumor invasion (*P* = 0.077), venous invasion (*P* = 0.157), and vertical margin (*P* = 0.228) had weak relationships. 

The results of the multivariate analysis are summarized in Table 3. Multivariate analysis showed that only venous invasion was significant with lymph node metastasis (*P* = 0.017). Age (*P* = 0.545), endoscopic treatment (*P* = 0.264), pathological tumor size (*P* = 0.482), depth of tumor invasion (*P* = 0.300), macro type (*P* = 0.121), lymphatic invasion (*P* = 0.269), and vertical margin (*P* = 0.482) were not significant. For remnant of the primary tumor, depth of tumor invasion (pT1b2 tumor invasion) (*P* = 0.015) and horizontal margin (*P* = 0.013) were significant. Sex (*P* = 0.365), endoscopic treatment (*P* = 0.071), pathological tumor size (*P* = 0.836), lymphatic invasion (*P* = 0.159), venous invasion (*P* = 0.091), and vertical margin (*P* = 0.072) were not significant.

## 4. Discussion

The most effective treatment for gastric cancer is surgery, including gastrectomy and lymphadenectomy [15]. However, surgery is often overtreatment, especially for EGC without nodal metastasis. If we estimate that the risk of nodal metastasis is very small, endoscopic treatment without lymphadenectomy (e.g., EMR and ESD) can be performed for EGC [16–18]. Depth of tumor invasion, ulceration, lymphatic and venous invasion, and undifferentiated component have been reported as risk factors of lymph node metastasis [4, 10]. When these risk factors are detected by pathological examination, surgical treatment with lymphadenectomy is generally added [11], despite the fact that the incidence of nodal metastases of EGC is rare [3]. EGC is a curable disease, but inadequate treatment may yield remnant metastatic lymph nodes. The Japanese Gastric Cancer Association created a criterion for safe endoscopic treatment [14]. As a result, some patients without nodal metastasis may undergo unnecessary surgical operations. 

In this study, the incidence of nodal metastases in tumors with non-curative potential after endoscopic resection was 9.8%. Statistically, only venous invasion was independently significant with nodal metastases in terms of pathological characteristics, and there was no nodal metastasis in the tumors without pathological venous invasion. Moreover, only 1 of 21 tumors without lymphatic invasion had nodal metastasis. In terms of the other pathological factors, mucosal tumors had no nodal metastasis, and ulceration and histological type had no significant relationship with nodal metastasis. Differences in risk factors for nodal metastasis exist between previous reports and the present study. These discrepancies were caused by differences in specimens. The risk factors of nodal metastases have been based on pathological findings of surgical specimens. In this study, we investigated specimens obtained by endoscopic resection. These findings should be confirmed in a large-scale study.

On the other hand, positive tumor involvement (HM1 and VM1) in endoscopically resected specimens is also generally considered to be an indication for additional endoscopic resection [13] or surgery [12, 13, 19]. However, the absence of pathological carcinoma in the surgical specimens was not rare. Oda et al. reported that 47.4% of surgical specimens containing tumors with a possible lateral margin had residual tumors [12]. It is thought that achieving an exact diagnosis with endoscopically resected specimens is sometimes difficult [20] because these specimens are histologically modified.

In our study, 3 of 4 (75.0%) patients with an HM1 tumor, 4 of 14 (28.6%) with a VM1 tumor, and 1 of 25 (4.0%) without evidence of margin involvement had a remnant of the primary tumor. In the statistical analysis, horizontal margin (*P* = 0.013) and pT1b2 tumor invasion (*P* = 0.015) were independently significant factors of remnants primary tumors. Vertical margin was not significant (*P* = 0.072). The endoscopic treatment, whether EMR or ESD, was also not significant. Because all 12 patients with remnants of primary tumors had no nodal metastases, it was suggested that tumor involvement is not always a necessary condition for additional lymphadenectomy. Thus, additional resection may be performed endoscopically. 

In our opinion, when non-curative endoscopic resection is considered, additional surgical resection including lymphadenectomy should be performed for tumors with venous invasion. If tumors with horizontal involvement have no venous invasion, endoscopic resection without lymphadenectomy may be allowed. Because 10% of submucosal tumors have nodal metastases, surgical resection including lymphadenectomy should be performed for tumors with vertical involvement and pT1b2 (submucosal invasion of ≥0.5 mm) tumors. 

## 5. Conclusions

We studied the clinicopathological data and surgical outcomes of 41 patients with potentially non-curative EGC after endoscopic resection. Reasons for surgery were risk of lymph node metastasis and incomplete tumor resection. Four and 6 tumors had lymph node metastases and remnants of primary tumors after additional curative surgery, respectively. In terms of the pathological characteristics of the endoscopically resected specimens, venous invasion was the only significant risk factor for nodal metastasis (*P* = 0.017). Tumors without venous invasion had no nodal metastasis. On the other hand, pT1b2 (submucosal invasion of ≥0.5 mm) tumor invasion (*P* = 0.015) and horizontal margin (*P* = 0.013) were significantly related to remnants of primary tumors. Sex, age, endoscopic treatment, pathological tumor size, histological type, ulceration, lymphatic invasion, and vertical margin had no significant relationship with either nodal metastasis or remnants of primary tumors. According to our data, surgery with limited lymphadenectomy is recommended for tumors with venous invasion or vertical involvement and pT1b2 tumors, and additional endoscopic resection may be allowed for tumors with horizontal involvement.

## Figures and Tables

**Table 1:** Clinicopathological findings of patients (*n* = 41).

Variables	Number of subjects (%)
Sex	
Male	38 (92.7)
Female	3 (7.3)
Endoscopic treatment	
Mucosal resection	9 (22.0)
Submucosal dissection	32 (78.0)
Surgical indication	
Submucosal tumor invasion	37 (90.2)
Lymphatic or venous invasion	27 (65.9)
Undifferentiated component^†^	24 (58.5)
Horizontal margin involvement	4 (9.8)
Vertical margin involvement	14 (34.1)
Surgical approach	
Laparoscopy	34 (82.9)
Hand assistance	4 (9.8)
Open laparotomy	3 (7.3)
Extent of gastrectomy	
Partial	5 (12.2)
Proximal	9 (22.0)
Distal	20 (48.8)
Total	7 (17.1)
Remnant of primary tumor (surgical specimen)	
No	35 (85.4)
Yes	6 (14.6)
Depth of tumor invasion^‡^	
pT1a	4 (9.8)
pT1b1	4 (9.8)
pT1b2	33 (80.5)
Lymph node metastasis (surgical specimen)	
pN0	37 (90.2)
pN1	4 (9.8)
TNM stage (surgical specimen)	
fStage IA	37 (90.2)
fStage IB	4 (9.8)

^†^Differentiated: well-differentiated adenocarcinoma, moderately differentiated adenocarcinoma, or papillary adenocarcinoma; undifferentiated: poorly differentiated adenocarcinoma, signet-ring cell adenocarcinoma, or mucinous adenocarcinoma.

^‡^According to the third English edition of the Japanese Classification of Gastric Carcinoma.

**Table 2:** Results of univariate analyses showing relationships among clinicopathological characteristics of specimens.

Variables	Lymph node metastasis		Remnant of primary tumor	
	Subject	*P* value	Subject	*P* value
Total	4/41 (9.8%)		6/41 (14.6%)	

Sex		0.729		0.386
Male	4/38 (10.5%)		5/38 (13.2%)	
Female	0/3		1/3 (33.3%)	
Age (years)		0.488		0.566
≤70	3/25 (12.0%)		4/25 (16.0%)	
>70	1/16 (6.3%)		2/16 (12.5%)	
Endoscopic treatment		0.355		0.015*
Mucosal resection	0/9		4/9 (44.4%)	
Submucosal dissection	4/32 (12.5%)		2/32 (6.3%)	
Tumor size (mm)		0.445		0.488
≤20	3/24 (12.5%)		3/24 (12.5%)	
>20	1/17 (5.9%)		3/17 (17.6%)	
Depth of tumor invasion^†^		0.404		0.077
pT1a–1b1	0/8		3/8 (37.5%)	
pT1b2	4/33 (12.1%)		3/33 (9.1%)	
Macrotype		0.156		0.566
Elevated	3/16 (18.8%)		2/16 (12.5%)	
Depressed	1/25 (4.0%)		4/25 (16.0%)	
Intratumoral ulceration or ulcer scar		0.582		0.852
No	4/36 (11.1%)		5/36 (13.9%)	
Yes	0/5		1/5 (20.0%)	
Main histologic type^‡^		0.517		0.639
Differentiated	4/35 (11.4%)		5/35 (14.3%)	
Undifferentiated	0/6		1/6 (16.7%)	
Undifferentiated component^‡^		0.578		0.672
No	3/27 (11.1%)		4/27 (14.8%)	
Yes	1/14 (7.1%)		2/14 (14.3%)	
Lymphatic invasion		0.284		0.307
L0	1/21 (4.8%)		2/21 (9.5%)	
L1	3/20 (15.0%)		4/20 (20.0%)	
Venous invasion		0.030*		0.157
V0	0/23		5/23 (21.7%)	
V1	4/18 (22.2%)		1/18 (5.6%)	
Horizontal margin^†^		0.582		0.001**
HM0 or HMX	4/37 (10.8%)		3/37 (8.1%)	
HM1	0/4		3/4 (75.0%)	
Vertical margin^†^		0.047*		0.228
VM0 or VMX	2/27 (7.4%)		2/27 (7.4%)	
VM1	2/14 (14.3%)		4/14 (28.6%)	

*P < 0.05, **P < 0.01.

^†^According to the third English edition of the Japanese Classification of Gastric Carcinoma.

^‡^Differentiated: well-differentiated adenocarcinoma, moderately differentiated adenocarcinoma, or papillary adenocarcinoma; undifferentiated: poorly differentiated adenocarcinoma, signet-ring cell adenocarcinoma, or mucinous adenocarcinoma.

**Table 3:** Results of multivariate analyses showing relationships among clinicopathological characteristics of the specimens.

Lymph node metastasis		Remnant of primary tumor	
Variables	*P* value	Variables	*P* value
Age	0.545	Sex	0.365
Endoscopic treatment	0.264	Endoscopic treatment	0.071
Pathological tumor size	0.482	Pathological tumor size	0.836
Depth of tumor invasion	0.300	Depth of tumor invasion	0.015*
Macrotype	0.121	Lymphatic invasion	0.159
Lymphatic invasion	0.269	Venous invasion	0.091
Venous invasion	0.017*	Horizontal margin	0.013*
Vertical margin	0.482	Vertical margin	0.072

*P < 0.05.

## Abbreviations

EGC: Early gastric cancer
UICC: International Union Against Cancer
JCG: Japanese Classification of Gastric Carcinoma
EMR: Endoscopic mucosal resection
ESD: Endoscopic submucosal dissection
HM: Horizontal margin
VM: Vertical margin.
